# Supplementary material for: Efficient Combination of Complex Chromatography, Molecular Docking and Enzyme Kinetics for Exploration of Acetylcholinesterase Inhibitors from Poria cocos
Source: Molecules. 2023 Jan 27;28(3):1228. doi: 10.3390/molecules28031228 (PMC9920314; doi:10.3390/molecules28031228)
Supplement: Supplementary file 1 [file molecules-28-01228-s001.zip › molecules-2101596-supplementary.pdf]

Table S1. Molecular docking analysis of potential acetylcholinesterase ligand in Huperzine A and *P.cocos*.

No.	Ligand name	Ligand structure	Binding energy (kcal•mol^{-1})	Interactions amino acid
1	Tumulosic acid	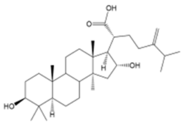	−7.77	PHE-288<br>ARG-289
2	Polyporenic acid C	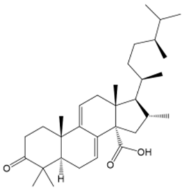	−7.88	PHE-288<br>SER-286<br>ARG-289
3	3-Epidehydrotu mulosic acid	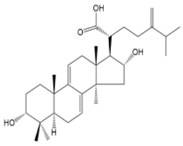	−7.37	PHE-288<br>ARG-289
4	Pachymic acid	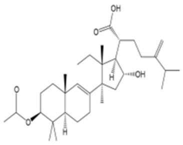	−8.01	PHE-288<br>ARG-289
5	Dehydro-trametenolic acid	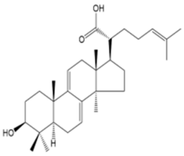	−6.92	PHE-288
6	Huperzine A	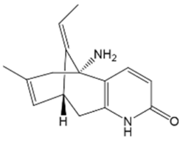	−7.51	SER-235

Table S2. Main features of AChE inhibition by active compounds.

Name	Compound 1	Compound 2	Compound 3	Compound 4	Compound 5
	Tumulosic acid	Polyporenic acid C	3-Epidehydrotum ulosic acid	Pachymic acid	Dehydro-trametenolic acid
^{1}H-NMR (400 MHz, $CDCl_3$) δ	δ: 1. 00 (3H, s, H-27), 1. 06 (3H, s, H-28), 1. 14	δ: 4.97, 4.88 (1H, s, H-31), 1.43 (3H, s, H-30), 1.11 (3H, s,	δ: 5.66 (1H, s, H-7), 5.53 (1H, d, *J* = 6.0 Hz, H-11),	δ: 4.98, 4.71 (1H, s, H-31), 4.06 (1H, t, *J* = 6.4 Hz, H-16),	δ: 5.33 (1H, d, *J* = 6.5 Hz, H-24), 1.65 (3H, s, H-26), 1.61 (3H, s,

	(3H, s, H-18), 0. 96, 0. 99 (3H, d, *J* = 6. 6 Hz, H-26, 27), 1. 24 (3H, s, H-18), 1. 45 (3H, s, H-30), 2. 32 (1H, m, H-25), 2. 94 (1H, d, *J* = 11. 4 Hz, H-17), 2. 95 (1H, m, H-20), 4. 55 (1H, t, *J* = 6. 0 Hz, H-16), 3. 44 (1H, t, *J* = 6. 0 Hz, H-3) 4. 81, 4. 97 (1H, s, H-31)	H-29), 1.12 (3H, s, H-19), 1.08 (3H, s, H-18), 1.06 (3H, s, H-28), 0.98, 0.96 (3H, d, *J* = 6.5 Hz, H-26, 27)	5.01 (1H, s, H-31), 4.59 (1H, t, J = 6.5 Hz, H-16), 2.97 (1H, m, H-20), 2.93 (1H, m, H-17), 2.33 (1H, m, H-25), 1.46 (3H, s, H-30), 1.22 (3H, s, H-28), 1.13 (3H, s, H-19), 1.07 (3H, s, H-18), 1.04, 0.96 (3H, d, *J* = 6.5 Hz, H-26, 27), 0.99 (3H, s, H-29)	2.33 (1H, m, H-25), 2.37 (1H, m, H-20), 1.74 (1H, dd, *J* = 11.2, 5.5 Hz, H-17), 1.54 (3H, s, H-30), 1.17 (3H, s, H-18), 1.12, 1.10 (3H, d, *J* = 6.4 Hz, H-26, 27), 1.02 (3H, s, H-28), 0.97 (3H, s, H-29), 0.98 (3H, s, H-19)	H-27), 1.20 (3H, s, H-28), 1.13 (3H, s, H-29), 1.04 (6H, s, H-19, 30), 0.99 (3H, s, H-18)
^{13}C-NMR (100 MHz, $CDCl_3$) δ	δ: 178. 3 (C-21), 156. 1 (C-24), 134. 4 (C-9), 135. 5 (C-8), 27. 4 (C-7), 21. 1 (C-11), 106.7 (C-31), 77. 8 (C-3), 76. 6 (C-16), 57. 2 (C-17), 49. 1 (C-14), 48. 8 (C-20), 46. 7 (C-13), 44. 5 (C-15), 51. 2 (C-5), 39. 6 (C-4), 37. 9 (C-10), 30. 2 (C-12), 34. 5 (C-25), 33. 5 (C-23)，31. 7 (C-22), 36. 8 (C-1), 29. 3 (C-28) 25. 6 (C-30), 29. 2 (C-2), 19. 5 (C-6), 17. 1 (C-29), 20. 1 (C-19), 22. 1 (C-26), 21. 8 (C-27), 17. 9 (C-18)	δ: 214.2 (C-3), 178.5 (C-21), 155.8 (C-24), 144.2 (C-9), 141.5 (C-8), 120.8 (C-7), 116.5 (C-11), 106.8 (C-31), 75.5 (C-16), 56.4 (C-17), 50.6 (C-5), 48.4 (C-14), 46.3 (C-4), 45.6 (C-13), 44.3 (C-15), 36.6 (C-10), 363 (C-12), 35.9 (C-1), 34.0 (C-25), 33.7 (C-23), 33.4 (C-2), 31.2 (C-22), 26.3 (C-29), 25.5 (C-30), 23.1 (C-6), 22.6 (C-20), 21.6 (C-26), 21.7 (C-19), 21.6 (C-27), 21.3 (C-28), 16.4 (C-18)	δ: 177.5 (C-21), 153.6 (C-24), 145.3 (C-9), 141.5 (C-8), 115.5 (C-11), 121.8 (C-7), 106.6 (C-31), 77.6 (C-16), 74.8 (C-3), 56.4 (C-17), 49.7 (C-20), 49.4 (C-14), 44.8 (C-13), 43.5 (C-5), 44.2 (C-15), 37.5 (C-12), 36.7 (C-10), 36.6 (C-4), 34.4 (C-25), 33.6 (C-23), 31.3 (C-22), 31.4 (C-1), 27.1 (C-28), 26.5 (C-30), 26.4 (C-2), 24.2 (C-6), 23.4 (C-29), 22.7 (C-19), 22.0 (C-26), 20.3 (C-27), 16.7 (C-18)	δ: 177.4 (C-21), 170.6 (CH 3 CO), 155.9 (C-24), 135.4 (C-8), 134.8 (C-9), 106.2 (C-31), 80.8 (C-3), 77.6 (C-16), 56.5 (C-17), 50.9 (C-5), 49.6 (C-20), 49.3 (C-14), 45.4 (C-13), 44.1 (C-15), 37.3 (C-4), 36.8 (C-10), 34.8 (C-1), 34.2 (C-25), 33.7 (C-23), 31.2 (C-22), 29.4 (C-12), 27.4 (C-28), 26.9 (C-7), 25.5 (C-30), 24.7 (C-2), 21.6 (C-26), 21.5 (C-27), 21.0 (C-11), 20.9 (CH 3 CO), 19.6 (C-19), 18.1 (C-6), 16.7 (C-18), 16.5 (C-29)	δ: 177.4 (C-21), 145.7 (C-9), 141.2 (C-8), 130.3 (C-25), 123.9 (C-24), 120.3 (C-7), 115.5 (C-11), 76.9 (C-3), 48.5 (C-5), 48.5 (C-20), 48.1 (C-14), 47.5 (C-17), 43.4 (C-13), 38.6 (C-4), 36.4 (C-10), 35.3 (C-1), 35.1 (C-12), 31.2 (C-22), 30.1 (C-15), 27.6 (C-2), 27.2 (C-28), 26.0 (C-16), 24.7 (C-23), 24.6 (C-26), 24.4 (C-30), 21.7 (C-6), 21.8 (C-19), 16.5 (C-27), 15.8 (C-29), 15.5 (C-18)

Table S3. Kinetic parameters of AChE inhibition by active compounds.

Active compound	Sample (μmol)	Michaelis-Menten equation	R^2	K_m /(K_m^{app})	V_m /(V_m^{app})	K_i (μM)	K_{is} (μM)
Pachymic acid	0	1/v = 885.6721/[S]+49.6839	0.9998	17.82	0.022	1.83	
	10	1/v = 3957.3928 / [S] + 49.7832	0.9980	65.91	0.022		

	15	1/v = 5539.1520 / [S] + 49.3591	0.9979	94.61	0.022		
	20	1/v = 7393.5920 / [S] + 49.6934	0.9946	132.94	0.022		
Polyporenic acid C	0	1/v = 885.6721 / [S] + 49.6839	0.9979	17.78	0.020		
	10	1/v = 1030.1048 / [S] + 59.5395	0.9835	17.65	0.017	51.47	
	15	1/v = 1145.8393 / [S] + 68.5693	0.9994	17.61	0.015		
	20	1/v = 1245.6549 / [S] + 73.7946	0.9835	17.47	0.011		
Tumulosic acid	0	1/v = 885.6721 / [S] + 49.6839	0.9914	17.82	0.020		
	10	1/v = 1323.1145 / [S] + 74.2773	0.9782	17.83	0.014	16.98	
	15	1/v = 1715.8828 / [S] + 95.9198	0.9976	17.79	0.010		
	20	1/v = 2839.2280 / [S] + 118.3527	0.9849	17.81	0.009		
3-Epidehydrotumulosic acid	0	1/v = 885.6721 / [S] + 49.6839	0.9859	17.82	0.020		
	10	1/v = 1323.1145 / [S] + 74.2773	0.9944	25.43	0.0087	3.17	7.21
	15	1/v = 1715.8828 / [S] + 95.9198	0.9902	30.63	0.0064	6.24	
	20	1/v= 2246.4298 / [S] + 118.3527	0.9834	17.81	0.0042		
Dehydro-trametenolic acid	0	1/v = 885.6721 / [S] + 49.6839	0.9975	17.82	0.020		
	10	1/v = 1857.6611 / [S] + 80.0579	0.9942	23.20	0.014	8.21	17.38
	15	1/v = 2969.9629 / [S] + 133.5642	0.9839	49.73	0.009	8.93	
	20	1/v = 4753.8244 / [S] + 49.6839	0.9834	93.57	0.020		
